# Psychopathic Traits and Psychological Violence in Intimate Partner Relationships: A Systematic Review of Observational Studies

**DOI:** 10.3390/healthcare14203499

**Published:** 2026-10-18

**Authors:** Laura Pérez-Díaz, Mónica Guerrero-Molina, Juan Manuel Moreno-Manso, Macarena Blázquez-Alonso

**Affiliations:** Department of Psychology, Faculty of Education and Psychology, University of Extremadura, 06006 Badajoz, Spain; laurapd@unex.es (L.P.-D.); jmmanso@unex.es (J.M.M.-M.); mablazqueza@unex.es (M.B.-A.)

**Keywords:** subclinical psychopathy, psychological violence, intimate partner violence, systematic review, psychopathic traits

## Abstract

**Background/Objectives**: Subclinical psychopathy is a relevant construct for the study of dysfunctional interpersonal dynamics and has been associated with different forms of violence in intimate relationships. The aim of this systematic review was to synthesise the available observational evidence on the association between psychopathic traits and psychological violence in intimate relationships, considering different psychopathy dimensions, manifestations of psychological violence, sources of information, and methodological characteristics of the studies. **Methods**: A literature search was conducted for studies published in English between January 2015 and March 2026 in Web of Science, Scopus, PsycINFO, and ScienceDirect, in accordance with the Preferred Reporting Items for Systematic Reviews and Meta-Analyses (PRISMA) guidelines. **Results**: Eight publications reporting nine independent studies, comprising a total of 2890 participants, were included. This aggregate figure represents the sum of the reported sample sizes and does not constitute a common analytical sample across studies. Data extraction covered sample characteristics, assessment instruments, psychopathy dimensions, manifestations of psychological violence, and main findings; methodological quality and risk of bias were assessed using the Newcastle–Ottawa Scale adapted for analytical cross-sectional studies (NOS-xs). Given the heterogeneity of measures, samples, and operationalisations, the findings were synthesised narratively. The reviewed studies showed variable associations between psychopathic traits and different manifestations of psychological violence, with differences according to the dimension assessed, sample type, source of information, and instrument used. In studies specifically examining Factor 1, affective–interpersonal traits were associated with some forms of psychological violence involving control, manipulation, and intimidation; other studies using global measures of psychopathy also identified associations with emotional–verbal abuse and dominance–isolation. Findings regarding Factor 2 were more heterogeneous across studies. Furthermore, some studies identified associations between psychopathy and psychological violence based on perpetrator self-reports, whereas others incorporated reports from victims or partners. **Conclusions**: Overall, the findings suggest an association between psychopathic traits and psychological violence in intimate relationships. However, the heterogeneity of the included studies and their exclusively cross-sectional design preclude causal inferences and warrant cautious interpretation of the observed associations. These findings may help inform hypotheses for future longitudinal and intervention research.

## 1. Introduction

In recent decades, subclinical psychopathy has received increasing attention in research on personality psychology and psychopathology owing to its implications for both intrapersonal functioning and interpersonal dynamics, as well as its potential to generate various forms of psychological harm within everyday social relationships [1,2]. Unlike the traditional conceptualisation of psychopathy associated with forensic settings, contemporary models advocate a dimensional perspective in which psychopathic traits are distributed along a continuum and may be present in individuals without a criminal history or significant clinical impairment.

From this perspective, subclinical psychopathy is understood as the moderate expression of such traits, manifested through affective, interpersonal, and behavioural patterns that are generally socially adaptive, yet nevertheless possess the potential to cause harm within interpersonal relationships [2,3].

This construct encompasses a range of characteristics, including emotional callousness, low empathy, a manipulative interpersonal style, social dominance, egocentrism, and a tendency towards impulsivity or sensation seeking, without necessarily reaching the levels of criminal behaviour typically associated with clinical psychopathy [1]. Although these traits are dimensionally distributed throughout the general population and may be expressed with varying degrees of intensity across individuals, their manifestation at subclinical levels may have important implications for interpersonal functioning. Although individuals displaying such traits may function in an apparently adaptive manner across academic, occupational, or social contexts, their dispositional characteristics have been associated with an increased likelihood of engaging in interpersonal relationships that are conflictual, unstable, or emotionally harmful.

In the context of intimate relationships, research has shown that individuals with high levels of subclinical psychopathy tend to establish relationships characterised by low intimacy, limited commitment, and a greater tendency to instrumentalise their partners [2,4]. These dynamics are associated not only with difficulties in forming affective bonds but also with the use of interpersonal control strategies involving manipulative and coercive behaviours. Consistent with these findings, interpersonal psychopathic traits, such as superficial charm and manipulativeness, have been linked to lower relationship quality, greater instability, and an increased likelihood of relationship dissolution [2].

In this context, psychological violence constitutes a particularly relevant focus of analysis and may be defined as a pattern of persistent behaviours involving control, manipulation, devaluation, intimidation, or emotional intrusion that undermines the victim’s self-esteem, emotional well-being, and psychological autonomy [5,6]. However, psychological aggression is not a homogeneous phenomenon and may range from isolated acts of psychological or verbal aggression, typically assessed using instruments focused on conflict tactics, to more sustained patterns of emotional abuse and coercive control, characterised by greater persistence, power asymmetry, and impairment of the partner’s autonomy. This distinction is relevant for appropriately interpreting the heterogeneity of the available findings. This form of violence, which is prevalent in intimate, familial, and occupational settings, is often especially difficult to identify owing to its gradual nature and its normalisation within relational dynamics. Several studies have indicated that core traits of subclinical psychopathy, including emotional coldness, lack of empathy, manipulativeness, and an instrumental view of relationships, are associated with a greater likelihood of perpetrating this form of interpersonal violence [7]. In particular, these characteristics have been linked to strategies such as gaslighting, emotional manipulation, and the exploitation of another person’s emotional vulnerabilities [4].

Evidence from the field of intimate partner relationships indicates that individuals exhibiting these profiles are more prone to coercive, passive-aggressive, and emotionally dominant behaviours, thereby contributing to the perpetuation of psychologically abusive relational dynamics [5,7]. Although these manifestations may share underlying mechanisms, they should not be considered equivalent in terms of severity, frequency, duration, or power imbalance. Outside the context of intimate relationships, dark personality traits have also been associated with harmful interpersonal behaviours in organisational settings; however, this evidence is included solely as general context and falls outside the scope of the present review.

From an integrative explanatory perspective, the relationship between subclinical psychopathy and psychological violence may be understood through multidimensional models that incorporate individual characteristics, emotional processes, and contextual factors underlying interpersonal relationships. In this regard, deficits in affective empathy, which are characteristic of this construct, may reduce the capacity for emotional engagement with others’ suffering, whereas the potential preservation or instrumental use of certain cognitive empathy abilities may facilitate the recognition of interpersonal vulnerabilities. However, this possibility represents a theoretical hypothesis rather than a causal pathway directly assessed in the studies included in the present review. The interaction of these processes may contribute to the emergence of psychological aggression through strategies involving emotional manipulation, interpersonal instrumentalisation, and interpersonal control [8].

These processes are complemented by cognitive mechanisms associated with psychopathic traits, such as alterations in moral decision-making, greater tolerance for interpersonal risk, and a tendency to prioritise personal benefit over the well-being of others [1]. These mechanisms should be considered as distinct potential theoretical explanations rather than as processes directly demonstrated by the included studies. In particular, impulsivity and reduced sensitivity to punishment may contribute to certain forms of aggressive behaviour, whereas interpersonal instrumentalisation and the prioritisation of personal benefit may be related to control strategies. However, the present review does not allow causal inferences to be drawn regarding whether these mechanisms account for psychological aggression.

Despite the growing interest in subclinical psychopathy and its association with various forms of interpersonal violence, available systematic reviews differ substantially in their conceptualisation of psychopathy, the manifestations of violence examined, and the populations studied. In particular, Azeredo et al. [9] conducted a systematic review with a meta-analytic approach examining the relationship between psychopathy and violence in intimate relationships, identifying 35 studies published between 1983 and 2024, of which 30 were included in the meta-analyses. This review provides a broad synthesis of the association between psychopathy and intimate partner violence across different forms of violence and operationalisations of psychopathy. The present review adopts a more specific scope by focusing on psychological aggression within intimate relationships and on dimensional or subclinical psychopathic traits.

In light of this evidence, there remains a need for a synthesis specifically focused on characterising the evidence on the association between psychopathic traits and psychological aggression in intimate partner relationships, while considering the heterogeneity of the measures employed and sources of information. The present review addresses this gap by searching for studies published between 2015 and March 2026 and applying criteria focused on the assessment of psychopathic traits and manifestations of psychological aggression in intimate relationships. This approach provides a more specific examination of the association between psychopathic traits and psychological aggression, while also allowing for a detailed assessment of the conceptual and methodological diversity of the included observational studies.

Furthermore, the available studies show divergent findings regarding the contribution of different dimensions of psychopathy. For example, whereas Iyican and Babcock [10] observed associations between Factor 1 and different forms of psychological aggression, with no significant associations for Factor 2, Plouffe et al. [11] identified associations between Factor 2 and greater severity of intimate partner violence. Similarly, Brassard et al. [12] distinguished between primary psychopathy, which was associated with severe psychological aggression, and secondary psychopathy, which was associated only with milder forms, whereas other studies based on global psychopathy scores or Dark Triad traits found associations with different manifestations of emotional abuse, dominance, or isolation [13,14]. These differences in the direction, specificity, and magnitude of the associations suggest that the relationship between psychopathic traits and psychological aggression may depend on the dimension assessed, the type of aggression considered, sample characteristics, and the source of information used.

These differences justify a new systematic synthesis aimed at identifying which dimensions of psychopathic traits are associated with different manifestations of psychological aggression in intimate partner relationships, how both constructs have been operationalised, and which methodological characteristics may help explain the heterogeneity of the findings.

In this context, the aim of the present systematic review is to examine the association between subclinical psychopathic traits and psychological violence in adolescents and adults who are currently or have previously been involved in intimate partner or intimate relationships. The exposure of interest comprises subclinical psychopathic traits, assessed across affective, interpersonal, and behavioural dimensions, while eligible studies compare different levels of these traits. Outcomes include different manifestations of psychological violence, both perpetrated and experienced, including emotional abuse, coercive control, manipulation, dominance, intimidation, and verbal aggression, recognising that these manifestations may differ in severity, frequency, duration, and degree of power imbalance. Studies based on self-reports, partner reports, or reports from other informants, and employing quantitative empirical cross-sectional or longitudinal designs, are also considered. Furthermore, the review aims to examine the conceptual definitions and assessment instruments used in the literature in order to provide an integrative overview of the current state of knowledge, assess the consistency of the available findings, and identify implications for future research and prevention and intervention strategies.

The present review examines the association between psychopathic traits and psychological violence in intimate partner relationships, focusing on studies published between January 2015 and March 2026.

## 2. Materials and Methods

This systematic review was reported in accordance with the Preferred Reporting Items for Systematic Reviews and Meta-Analyses (PRISMA) 2020 statement [15] (Supplementary Material Table S1: PRISMA Checklist). The methodological procedures used to conduct the review are described separately in the following sections, including the eligibility criteria, literature search, study selection, methodological quality assessment, data extraction, and evidence synthesis.

### 2.1. Protocol and Registration

The methodological procedures for this systematic review, including the eligibility criteria, search strategy, study selection, data extraction, and methodological assessment, were agreed upon by the authors before the review process was conducted. However, no separate written protocol was prepared, and the review was not prospectively registered in a protocol registration platform. Consequently, no registration number or registration date is available.

### 2.2. Selection Criteria

To be included in the present systematic review, publications were required to meet the following inclusion criteria: (a) empirical studies examining subclinical psychopathy and its relationship with psychological violence; (b) studies employing samples comprising participants in developmental stages equivalent to or beyond early adolescence (approximately 13 years of age), a period associated with the normative onset of romantic relationships; (c) studies examining psychological violence within intimate partner or romantic relationships, including current or former relationships; (d) studies specifically addressing psychological violence as a distinct construct; (e) studies that use established and psychometrically validated instruments; (f) studies explicitly reporting the results derived from the assessment of the variables under investigation; (g) studies providing sufficient information to determine the presence of subclinical psychopathy and psychological violence, including its specific manifestations when these were conceptualised and assessed as components of psychological violence, thereby enabling the analysis and interpretation of the association between both constructs within the sample; and (h) studies published between 2015 and 2026.

The following exclusion criteria were also applied: (a) publications that, following title and abstract screening, did not provide sufficient evidence of relevance to the predefined constructs of subclinical psychopathy and psychological violence or their corresponding manifestations; (b) studies focused exclusively on criminal psychopathy or conducted exclusively in correctional settings; (c) publications that did not specify the type of psychopathy or psychological violence assessed, or in which these variables were mentioned but were not examined as relevant constructs within the scope of the study; and (d) studies in which psychopathy or psychological violence could not be identified as distinct constructs or in which the reported data did not allow the association between these constructs to be examined separately from unrelated constructs or outcomes.

### 2.3. Search Strategy

To identify relevant studies, a comprehensive search of the scientific literature published in English between January 2015 and March 2026 was conducted. This period was defined to provide a clearly delimited timeframe for the identification and synthesis of recent empirical evidence concerning subclinical psychopathic traits and psychological violence within intimate partner relationships. English was selected as the search language because it is the predominant language of international scientific communication and is widely used for the dissemination of research in psychology, behavioural sciences, forensic research, and related health and social science fields.

The databases Web of Science, Scopus, ScienceDirect, and PsycINFO were searched using a predefined combination of terms related to subclinical psychopathy and psychological violence, linked through Boolean operators as follows: (“integrated psychopathy” OR “subclinical psychopathy” OR “psychopathic traits”) AND (“psychological violence” OR “psychological abuse” OR “psychological maltreatment”). The Boolean search strategy was applied to each database using the search fields available in the respective platforms. In Web of Science, the terms were searched using the Topic field; in Scopus, the search was conducted across article title, abstract, and keywords; in PsycINFO via EBSCO, the “All Fields” option was used; and in ScienceDirect, the general search function was used. The only filter applied was publication year, restricted to studies published between January 2015 and March 2026. The searches were conducted on 12 April 2026. The publication cut-off was set at March 2026 to ensure that the eligibility period comprised complete calendar months and to avoid including only the first 12 days of April 2026. The database-specific search fields, search strategies, applied limits and search date are reported in Supplementary Table S2.

The search and selection criteria were jointly established by Macarena Blázquez-Alonso, Mónica Guerrero-Molina, and Laura Pérez-Díaz Macarena Blázquez-Alonso, Mónica Guerrero-Molina, and Laura Pérez-Díaz independently screened each title and abstract according to the predefined inclusion and exclusion criteria. Potentially eligible articles then underwent full-text assessment by Macarena Blázquez-Alonso, Mónica Guerrero-Molina, Laura Pérez-Díaz, and Juan Manuel Moreno-Manso, who independently evaluated each article against the eligibility criteria. Any disagreements during title and abstract screening were resolved through discussion among the three reviewers involved. Disagreements during full-text assessment were resolved through discussion among the reviewers involved and, where necessary, by Macarena Blázquez-Alonso when Juan Manuel Moreno-Manso had participated in the initial assessment.

### 2.4. Quality Assessment and Risk of Bias

The methodological quality of the included studies was independently assessed by reviewer pairs (Macarena Blázquez-Alonso/Laura Pérez-Díaz and Mónica Guerrero-Molina/Juan Manuel Moreno-Manso), using the Newcastle–Ottawa Scale adapted for analytical cross-sectional studies (NOS-xs) developed by Carra et al. [16]. This tool was selected because all studies included in the present review employed a cross-sectional design, examining associations between psychopathic traits and psychological violence. The NOS-xs was specifically developed to assess the risk of bias of analytical cross-sectional studies and represents an adaptation of the original Newcastle–Ottawa Scale, which was designed for cohort and case–control studies [16].

The NOS-xs evaluates six items distributed across three domains: study sample selection, assessment of exposure(s) and outcome(s), and confounding factors. The first domain evaluates the representativeness of the study sample and sample size; the second assesses exposure and outcomes; and the third assesses adjustment for confounding factor(s). Each item is rated according to predefined criteria and contributes a maximum of one or two stars, resulting in a total score ranging from 0 to 9 stars. Higher scores indicate a lower risk of bias. Following the criteria established by Carra et al. [16], studies scoring 7–9 stars were classified as having a low risk of bias, those scoring 4–6 stars as having a moderate risk of bias, and those scoring 0–3 stars as having a high risk of bias [16].

Any discrepancies arising between reviewers were resolved through discussion. When Juan Manuel Moreno-Manso was involved in the initial assessment, disagreements were resolved by Macarena Blázquez-Alonso, who was not involved in the original assessment of the corresponding study. Macarena Blázquez-Alonso provided overall methodological supervision of the assessment process.

### 2.5. Data Extraction and Analysis

Data from the eligible studies were extracted and analysed independently by pairs of reviewers (Macarena Blázquez-Alonso/Laura Pérez-Díaz and Mónica Guerrero-Molina/Juan Manuel Moreno-Manso). Relevant variables relating to methodological design and sample characteristics were collected, including: (a) the country in which the study was conducted; (b) the research design; (c) analytical approach; (d) sample-specific reliability coefficients; (e) the sample size; (f) participants’ gender and age; (g) the instruments used to assess subclinical psychopathy and psychological violence; and (h) the findings obtained from the assessment of subclinical psychopathy and psychological violence.

The extracted information was systematically recorded in structured data-extraction tables. In cases of disagreement between reviewers, discrepancies were resolved through discussion and consensus among the reviewers involved. Macarena Blázquez-Alonso provided overall methodological supervision of the data extraction process. The numerical results were checked against the corresponding primary publications, and when discrepancies or uncertainties were identified, the original publication was consulted and the information reported in the primary study was retained.

Given the substantial heterogeneity across the included studies in the conceptualisation and operationalisation of subclinical psychopathy and psychological violence, as well as in the assessment instruments, sample characteristics, outcome definitions, and statistical approaches, a quantitative meta-analysis was not considered appropriate. Although several studies reported correlation coefficients, regression coefficients, or other measures of association, these estimates were not sufficiently comparable to support a valid pooled effect estimate. Therefore, a structured narrative synthesis was conducted. The findings were compared according to the psychopathy dimensions assessed, the type of psychological violence examined, the direction of the association, the characteristics of the study samples, and the statistical methods used. The consistency and direction of the reported associations were considered across studies, together with the magnitude and precision of effect estimates where available, the adjustment for relevant covariates, and the methodological quality of each study. No statistical weighting of studies was applied. Statistical significance was not used as the sole criterion for determining the consistency or relevance of findings.

When individual studies reported multiple relevant findings, results were extracted and organised according to a structured hierarchy aligned with the review question. First, findings concerning psychopathic traits were considered, including both global psychopathy measures and specific dimensions or factors when these represented the operationalisation of psychopathy used in the study. Second, findings concerning psychological violence were considered, including the overall construct and specific manifestations when these were explicitly conceptualised and assessed as components of psychological violence. Third, findings directly examining the association between psychopathic traits and psychological violence were prioritised as the primary evidence of interest. Fourth, results obtained from different informants or assessment approaches were retained separately when they represented distinct assessments of the association between psychopathic traits and psychological violence. Fifth, when studies reported multiple statistical estimates addressing the same association, all estimates directly relevant to the review question were considered. Findings concerning physical violence, sexual abuse, financial abuse, property damage, physical injury, or general offending were extracted when reported but were treated as contextual findings rather than primary outcomes. This approach was used to ensure consistent extraction and synthesis of the available evidence and to minimise selective emphasis on individual findings.

Reference management was conducted using Mendeley Reference Manager (version 2.144.0), which was used to organise the retrieved records, manage bibliographic references, and assist in the identification and removal of duplicate records during the study-selection process.

## 3. Results

### 3.1. Selection of the Studies and Their Characteristics

**Figure 1:**
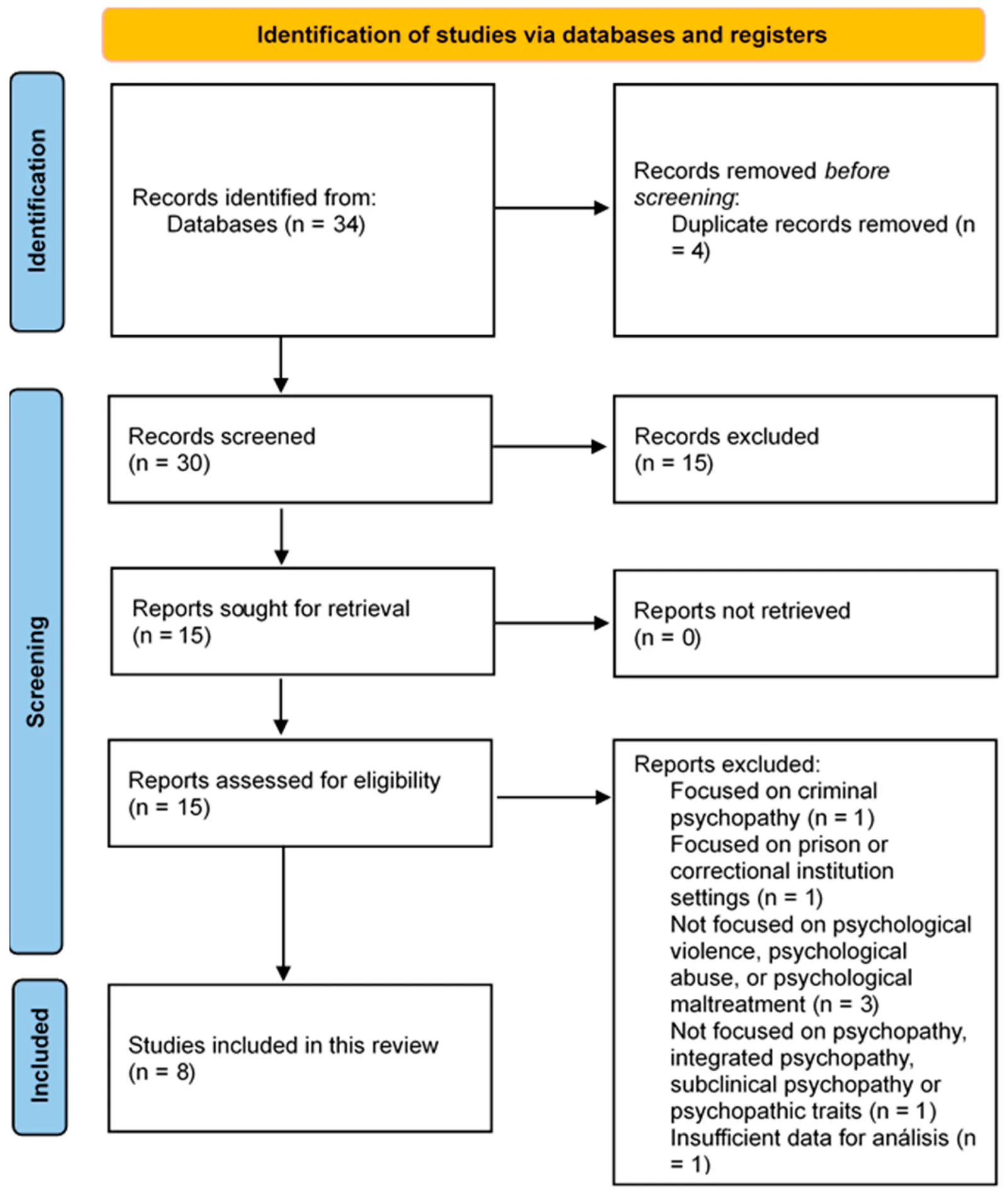
PRISMA flow diagram. Adapted from Page et al. [15].

The database search yielded a total of 34 articles, comprising 11 records from Web of Science, three from Scopus, 16 from ScienceDirect, and four from PsycINFO. First, four duplicate records were removed, leaving 30 records for title and abstract screening. During the screening stage, 15 records were excluded. In total, 15 articles addressing subclinical psychopathy and psychological violence were identified. However, following full-text assessment, a further seven articles were excluded for failing to meet the predefined inclusion and exclusion criteria. The specific reasons for exclusion for each study are presented in Table 1. Finally, eight studies [10,11,12,13,14,17,18,19] were included in the review. One of these publications, Plouffe et al. [11], reports two independent studies with separate samples and analytical procedures. Thus, the eight included publications correspond to nine independent studies. Details of the selection process are presented in the PRISMA flow diagram [15] shown in Figure 1.

The studies included in the present review exhibited a relatively concentrated geographical distribution, with a predominance of research conducted in Canada (37.5%) [11,12,13]. Additional studies were identified from Australia (12.5%) [17], Italy (12.5%) [14], the United States (12.5%) [10], and the United Kingdom (12.5%) [19]. In one of the included studies, the country of origin was not specified [18] (12.5%).

### 3.2. Study Design and Variables Examined in the Included Studies

Table 2 summarises the main characteristics of the studies included in this review. Table 3, in turn, provides a detailed overview of sample characteristics, the assessment instruments employed, the principal empirical findings, and the analytical approaches used across studies. Although all included studies used a cross-sectional design, providing a shared temporal framework, substantial clinical and methodological heterogeneity was observed across studies. This heterogeneity concerned sample characteristics, sources of information, assessment instruments, operationalisation of psychopathic traits and psychological violence, and statistical approaches used to examine their associations.

**Table 2:** Characteristics of the studies included in this review.

Authors	Publication Year	Country	Design	Analytical Approach	Reliability Coefficients and Sources
Brassard et al. [12]	2022	Canada	Cross-sectional study	Multiple regression and logistic regression analyses	CTS-2: α = 0.84 (minor physical), 0.75 (minor psychological), 0.71 (minor sexual), 0.89 (severe physical), 0.69 (severe psychological), 0.93 (severe sexual); ECR: α = 0.90 (anxiety), 0.81 (avoidance); LSRP: α = 0.77 (primary psychopathy), 0.71 (secondary psychopathy)
Brazil & Forth [13]	2024	Canada	Cross-sectional study	Descriptive statistics, bivariate correlations, hierarchical multiple regression, and mediation analyses	PCL:YV: α = 0.93 (total; facets α = 0.80–0.88); PMWI: α = 0.80 (DI), 0.89 (EV); CTS: α = 0.72
Bui & Pasalich [17]	2021	Australia	Cross-sectional study	Saturated path analysis/mediation with FIML estimation and bias-corrected bootstrapping	ECR-R (Attachment Anxiety): α = 0.92; ECR-R (Attachment Avoidance): α = 0.92; MBPD: α = 0.73; LSRP: α = 0.85; MMEA: α = 0.89; CPA: α = 0.79
Fontanesi et al. [14]	2024	Italy	Cross-sectional study	Descriptive statistics, t-tests, Pearson correlations, and stepwise multiple linear regression analyses	DD: α = 0.80 (narcissism), 0.67 (psychopathy), 0.84 (Machiavellianism), 0.85 (total); ASP: α = 0.71; IPVAS-R: α = 0.67–0.75; MJS: α = 0.60–0.90; MMEA: α = 0.86
Humeny et al. [18]	2021	Not reported	Cross-sectional study	Point-biserial correlations with Bonferroni correction, simple linear regression, and multiple regression analyses	MSRP-SF: α = 0.85 (total), 0.78 (Factor 1), 0.73 (Factor 2), 0.75 (IM), 0.67 (EL), 0.60 (AB), 0.60 (CA)
Iyican & Babcock [10]	2018	United States	Cross-sectional study	Pearson correlations and hierarchical multiple regression analyses	PPI-SF: α = 0.77 (PPI-I), 0.82 (PPI-II); CTS-2: α = 0.60–0.78
Plouffe et al. [11]	2022	Canada	Cross-sectional study	Study 1: Descriptive statistics, reliability analyses, Spearman and Pearson correlations, and negative binomial regression with interaction analyses Study 2: Descriptive statistics, reliability analyses, Spearman and Pearson correlations, and ordinal logistic regression with interaction analyses	SRP-III: α = 0.89–0.92 (reported in previous research); NPI: α = 0.87 (reported in previous research); MACH-IV: α = 0.83 (reported in previous research); ASP: α = 0.83 (reported in previous research); CTS-2: α = 0.79–0.95 (reported in previous research)
Thornton et al. [19]	2016	United Kingdom	Cross-sectional study	MANOVA, Pearson correlations, and negative binomial regression analyses	YPI: α = 0.93 (Factor 1), 0.83 (Factor 2); NVOBS: α = 0.79 (IPV), 0.89 (general violence), 0.71 (nonviolent offending); BPAQ (anger): α = 0.83; BSCS: α = 0.80

**Table 3:** Sample Characteristics, Assessment Instruments, and Main Findings of the Studies Included in This Review.

Authors	Sample	Gender and Age	Instruments	Findings
Brassard et al. [12]	*n* = 226	Men = 226 (100%) Mean age = 34.18 years	LSRP CTS-2 ECR	Secondary psychopathy was associated with greater perpetration of mild psychological violence within intimate partner relationships (standardized regression coefficient, *β* = 0.173; *p* = 0.024; squared semi-partial correlation, *sr*^2^ = 0.020), whereas primary psychopathy (standardized regression coefficient, *β* = 0.045; *p* = 0.496) was not significantly associated with this form of violence. In contrast, primary psychopathy (standardized regression coefficient, *β* = 0.189; *p* = 0.007; *sr*^2^ = 0.032), but not secondary psychopathy (*β* = 0.115; *p* = 0.149), was significantly associated with more frequent perpetration of severe psychological violence against women.
Brazil & Forth [13]	*n* = 156 Participants recruited from secure custody facilities (*n* = 106) Participants recruited from community settings (*n* = 50)	Men = 156 (100%) Mean age = 17.44 years	Semi-structured interview PCL:YV PMWI CTS	Psychopathic traits were associated with all measures of intimate partner violence, including dominance–isolation, emotional–verbal abuse, and physical abuse, with the corresponding models explaining 7%, 8%, and 3% of the variance in each measure, respectively. However, the associations between dominance–isolation, emotional–verbal abuse, and psychopathic traits remained robust after controlling for sample type, whereas the association between physical abuse and psychopathic traits may be explained by the higher scores observed among adolescents in custody. After controlling for the effects of age and sample type, indirect associations were observed between exposure to interparental violence and dominance–isolation abuse through psychopathic traits (unstandardized indirect effect, *b* = 0.06, *SE* = 0.03; *β* = 0.04, 95% CI (0.01–0.09)), as well as between interparental violence and emotional–verbal abuse through psychopathic traits (unstandardized indirect effect, *b* = 0.10, *SE* = 0.05; *β* = 0.05, 95% CI (0.01–0.10)).
Bui & Pasalich [17]	*n* = 200	Women = 147 (73.5%) Men = 53 (26.5%) Mean age = 22.3 years	ECR-R MBPD LSRP MMEA CPA	Psychopathic traits and borderline personality traits were positively associated with the perpetration of both face-to-face psychological violence (standardized regression coefficients, *β* = 0.27 and *β* = 0.32, respectively; *p* < 0.001) and cyber psychological violence (standardized regression coefficients, *β* = 0.24 and *β* = 0.29, respectively; *p* < 0.001). Statistically significant indirect associations were observed between insecure attachment (anxiety and avoidance) and psychological violence through psychopathic traits. For face-to-face psychological violence, the indirect associations were *B* = 1.58, *SE* = 0.83, 95% CI (0.39–3.61) for attachment anxiety and *B* = 1.39, *SE* = 0.66, 95% CI (0.26–2.92) for attachment avoidance. For cyber psychological violence, the corresponding indirect associations were *B* = 0.65, *SE* = 0.32, 95% CI (0.19–1.45) and *B* = 0.57, *SE* = 0.30, 95% CI (0.10–1.31), respectively. Attachment anxiety also showed a statistically significant indirect association with psychological violence through borderline personality traits. No significant gender differences were observed.
Fontanesi et al. [14]	*n* = 491	Women = 253 (52%) Men = 238 (48%) Mean age = 32.95 years	Sociodemographic questionnaire DD IPVAS-R MJS MMEA	Men obtained higher scores on Dark Triad personality traits (*t* = 5.67; *p* < 0.001), sadism (*t* = 7.70; *p* < 0.001), and overall intimate partner violence (*t* = 4.52; *p* < 0.001), whereas emotional abuse, perpetrated in the form of restrictive intimidation and denigration, was more frequently reported among women (*t* = −3.84, *p* < 0.001 and *t* = −2.49, *p* < 0.05, respectively). Furthermore, psychopathy was positively associated with psychological abuse (*r* = 0.224, *p* < 0.01). The overall Dark Triad score was also positively associated with the total score on the Intimate Partner Violence Attitude Scale (*r* = 0.285, *p* < 0.01) and with partner control (*r* = 0.094, *p* < 0.05). The correlation between the overall Dark Triad score and physical violence was not statistically significant.
Humeny et al. [18]	*n* = 475	Women = 475 (100%) Mean age = 45.2 years	MSRP-SF Victimization Screening Survey	Survivors attributed psychopathic traits to their partners (*M* = 99.7; *SD* = 19.5), with a higher average level of Factor 1 (affective–interpersonal) (*M* = 53.8; *SD* = 10.5) than Factor 2 traits (behavioural–antisocial) (*M* = 43.4; *SD* = 11.5). At the facet level, interpersonal manipulativeness (*M* = 28.7) and egocentric lifestyle (*M* = 28.7) were particularly prominent, whereas antisocial behaviour (*M* = 25.1) showed the lowest scores. Abuse was described as frequent and characterised by a high degree of polyvictimisation, with victims experiencing, on average, more than five distinct forms of abuse. In this selected sample of survivors of intimate partner abuse, emotional abuse was reported by 99.6% of participants, deception by 95.3%, physical abuse by 62.4%, and sexual abuse by 59.9%. Psychopathic traits were significantly associated with multiple forms of intimate partner abuse, including physical abuse (*r* = 0.29), sexual abuse (*r* = 0.20), financial abuse (*r* = 0.29), and property damage (*r* = 0.28). Psychopathy accounted for 5% of the variance in abuse frequency (R^2^ = 0.05), 8% in physical injury (R^2^ = 0.08), and 14% in abuse versatility (R^2^ = 0.14). Factor 2 was associated with physical harm, whereas Factor 1 was associated with manipulative forms of abuse.
Iyican & Babcock [10]	*n* = 228	114 heterosexual couples (228 individuals) Women = 114 (50%) Men = 114 (50%) Age = 18 years and older 97 couples reported at least one incident of male-to-female violence in the previous year; 17 distressed but nonviolent couples were recruited as part of the larger study	PPI-SF CTS-2	All forms of male intimate partner violence self-reported by men were significantly correlated with Factor 1 psychopathy scores, with correlations ranging from *r* = 0.22 to *r* = 0.40 (*p* < 0.05). When male intimate partner violence reported by women was used, only severe physical violence was significantly correlated with Factor 1 scores (*r* = 0.20, *p* < 0.05). None of the forms of male intimate partner violence, whether self-reported by men or reported by women, was significantly correlated with Factor 2, relating to impulsivity and lifestyle.
Plouffe et al. [11]	Study 1 (*n* = 399) Study 2 (*n* = 360)	Study 1 Women = 290 (72.7%) Men = 109 (27.3%) Mean age = 18.74 years Study 2 Women = 207 (57.5%) Men = 153 (42.5%) Mean age = 34.39 years	SRP-III NPI MACH-IV ASP CTS-2	Study 1 Factor 1 and Factor 2 psychopathic traits were correlated with both psychological and physical aggression. Bidirectional aggression intensified at lower levels of psychopathy and Machiavellianism. Participants’ psychological aggression was associated with partner aggression (5%), narcissism (2%), and sadism (20%). Significant interactions indicated that bidirectional aggression was stronger at lower levels of Factor 1 and Factor 2 psychopathy (IRR = 1.04–1.07) and Machiavellianism (IRR = 1.04–1.06; all *p* < 0.001). No significant interactions were observed for narcissism or sadism. Study 2 Factor 2 psychopathy was associated with greater severity of intimate partner violence perpetration. Ordinal logistic regression analyses indicated that being female (*b* = 0.84–1.03; OR = 2.33 (95% CI: 1.51–3.57) to 2.81 (95% CI: 1.76–4.46)), greater IPV victimisation (*b* = 0.15–0.17; OR = 1.16 (95% CI: 1.08–1.25) to 1.19 (95% CI: 1.11–1.28)), and Factor 2 psychopathy (*b* = 0.65–0.84; OR = 1.91 (95% CI: 1.25–2.92) to 2.31 (95% CI: 1.33–4.02)) were associated with more severe perpetration. Interactions involving narcissism, sadism, Machiavellianism, and Factor 1 psychopathy were not statistically significant. Consistent findings were obtained using multiple linear regression analyses.
Thornton et al. [19]	*n* = 355	Women = 171 (48%) Men = 184 (52%) Mean age = 21.75 years	YPI NVOBS BPAQ BSCS	Men scored significantly higher than women on Factor 1 psychopathic traits, which assess interpersonal deficits (*F* = 50.79; Cohen’s *d* = 0.80), and Factor 2 psychopathic traits, which assess an impulsive and irresponsible lifestyle (*F* = 11.44; Cohen’s *d* = 0.38), with medium to large effect sizes. Both psychopathy factors were associated with intimate partner violence in both sexes (Men: Factor 1, *r* = 0.35; Factor 2, *r* = 0.26, *p* < 0.001; Women: Factor 1, *r* = 0.27; Factor 2, *r* = 0.27, *p* < 0.001), although they were also associated with the perpetration of general violence and non-violent offending. Factor 1 psychopathy was associated with general violence (χ^2^ = 5.66; *p* < 0.05) and non-violent offending (χ^2^ = 5.31; *p* < 0.05), although it was not significantly associated with intimate partner violence. In contrast, Factor 2 was not significantly associated with any type of offending.

Abbreviations: LSRP = Levenson Self-Report of Psychopathy; CTS-2 = Conflict Tactics Scale-2; ECR = Experiences in Close Relationships; PCL:YV = Psychopathy Checklist: Youth Version; PMWI = Psychological Maltreatment of Women Inventory; CTS = Conflict Tactics Scale; ECR-R = Experiences in Close Relationships-Revised; MBPD = Minnesota Borderline Personality Disorder; MMEA = Multidimensional Measure of Emotional Abuse; CPA = Cyber Psychological Abuse; DD = Dark Triad Dirty Dozen; IPVAS-R = Intimate Partner Violence Attitude Scale; MJS = Multidimensional Jealousy Scale; MSRP-SF = Modified Self-Report Psychopathy Scale-Short; PPI-SF = Psychopathic Personality Inventory–Short Form; SRP-III = Self-Report Psychopathy Scale III; NPI = Narcissistic Personality Inventory; MACH-IV = Machiavellianism Scale-IV; ASP = Aggression Subscale of the Physical Aggression Scale; YPI = Youth Psychopathic Traits Inventory; NVOBS = Non-Violent and Violent Offending Behavior Scale; BPAQ = Anger subscale of Buss–Perry aggression questionnaire; BSCS = Brief self-control scale. Note: sr^2^ = squared semi-partial correlation, indicating the proportion of unique variance in the outcome associated with a predictor after accounting for the other predictors in the model. Note: Psychological violence was the primary outcome of the present systematic review. Findings concerning physical violence, sexual abuse, financial abuse, property damage, physical injuries, and general offending are reported as contextual findings and were not included as primary outcomes in the synthesis.

In all of the studies included (100%) [10,11,12,13,14,17,18,19], the research was conducted in community, educational, or security-related settings, and participants were recruited from institutional records or voluntary samples. In Iyican and Babcock [10], the final sample comprised 114 heterosexual couples, including 97 couples who reported at least one incident of male-to-female violence in the previous year and 17 distressed but nonviolent couples recruited as part of the larger study. Most studies examined psychopathic traits dimensionally, using scores that allowed variation in trait levels to be examined within the study samples. Accordingly, the relevant methodological consideration is the extent of variability in psychopathic traits and psychological violence outcomes within the study samples and whether this variability was sufficient to examine their association.

### 3.3. Number, Gender, and Age of Participants

Across the reported samples of the included publications, a total of 2890 participants were included, with sample sizes ranging from a minimum of 156 [13] to a maximum of 491 [14]. Of these, 1657 (57.3%) were women and 1233 (42.7%) were men. These aggregated figures are provided for descriptive purposes only and should not be interpreted as representing a homogeneous analytical population. Gender distribution varied across studies: two investigations included exclusively male participants [12,13], one was conducted solely with women [18], and the remaining five employed mixed-gender samples, with the proportion of women ranging from 48% to 73.5% and that of men from 26.5% to 52% [10,11,14,17,19].

The reported sample sizes represent the individuals included in the respective study samples and should not be interpreted as a common pooled analytical sample. In most studies, the reported sample size corresponded to the participants who contributed data to the relevant analyses. However, analytical units and informants differed according to study design. For example, Iyican and Babcock [10] included 114 heterosexual couples (228 individuals), but psychopathy was assessed only in the 114 male participants. Male participants provided self-reports of intimate partner violence (IPV) perpetration, whereas female partners provided reports of male-to-female IPV. Thus, although 228 individuals were included in the sample, the psychopathy variable was based on 114 men. Similarly, Humeny et al. [18] included 475 participants who reported on psychopathic traits attributed to their abusive intimate partners; nine cases were subsequently excluded from the psychopathy measure, leaving 466 participants for the analyses. Accordingly, the aggregate sample of 2890 represents the total number of participants included across the reported samples, rather than a single analytical sample.

Participants’ mean age showed considerable variation across studies, ranging from 17.44 years in the sample reported by Brazil and Forth [13] to 45.2 years in the sample examined by Humeny et al. [18].

### 3.4. Assessment Instruments for Subclinical Psychopathy and Psychological Violence

Information regarding the instruments employed in the included studies is presented in Table 3. In total, the studies utilised 25 unique instruments to assess psychopathic traits, psychological violence, and other variables related to attachment, personality disorders, and characteristics of interpersonal relationships. Instruments used in more than one study were counted only once in determining the number of unique instruments. Distinct versions of the same measure were counted separately when they differed in item structure, scoring procedures, or assessment format (e.g., CTS and CTS-2; ECR and ECR-R). Among the most frequently used instruments were the Conflict Tactics Scale-2 (CTS-2), which was used in three studies, the Levenson Self-Report Psychopathy Scale (LSRP), which was used in two studies, and the Multidimensional Measure of Emotional Abuse (MMEA), which was used in two studies.

The included studies assessed psychopathic traits using a range of instruments. Six instruments were specifically designed to assess psychopathy or psychopathic traits: the Levenson Self-Report of Psychopathy (LSRP), Psychopathy Checklist: Youth Version (PCL: YV), Modified Self-Report Psychopathy Scale-Short (MSRP-SF), Psychopathic Personality Inventory–Short Form (PPI-SF), Self-Report Psychopathy Scale III (SRP-III), and Youth Psychopathic Traits Inventory (YPI). These instruments assessed core dimensions of psychopathic traits, including emotional callousness, lack of empathy, interpersonal manipulativeness, egocentrism, impulsivity, emotional dysregulation, risk-taking behaviours, and antisocial tendencies. The Dark Triad Dirty Dozen (DD) was not classified as a psychopathy-specific instrument because it assesses the broader Dark Triad construct, comprising narcissism, Machiavellianism, and psychopathy.

For the assessment of psychological violence and emotional abuse, five instruments were identified: the Conflict Tactics Scale-2 (CTS-2), the Psychological Maltreatment of Women Inventory (PMWI), the Conflict Tactics Scale (CTS), the Multidimensional Measure of Emotional Abuse (MMEA), and the Cyber Psychological Abuse (CPA) measure. These instruments assessed different forms of psychological aggression, psychological maltreatment, and emotional abuse within interpersonal relationships, including verbal aggression, humiliation, emotional manipulation, devaluation, intimidation, and other forms of overt and covert psychological abuse. Other instruments assessed related constructs, including attachment, attitudes toward intimate partner violence, jealousy, personality traits, aggression, offending behaviour, and self-control.

### 3.5. Risk of Bias Assessment

The risk of bias of the eight publications included in the present systematic review was assessed using the Newcastle–Ottawa Scale adapted for analytical cross-sectional studies (NOS-xs) [16]. For Plouffe et al. [11], the risk of bias was assessed separately for Study 1 and Study 2, given that they involved independent samples and analytical procedures. The results of the assessment for each study and each NOS-xs item are presented in Table 4.

Overall, the included studies obtained 7 stars out of 9 according to the predefined NOS-xs criteria. The individual item-level assessments presented in Table 4 allow potential methodological limitations across the different domains to be considered alongside the overall scores.

### 3.6. Findings from the Assessment of Subclinical Psychopathy and Psychological Violence

The primary outcome of this systematic review was the association between subclinical psychopathic traits and psychological violence within intimate partner relationships. Although some included studies also reported findings concerning other forms of violence or aggressive and offending behaviours, these findings are presented below as contextual evidence and were not considered primary outcomes of the present review. The synthesis of the primary findings therefore focuses specifically on psychological violence and its relationship with subclinical psychopathic traits.

#### 3.6.1. Subclinical Psychopathic Traits

Subclinical psychopathic traits were assessed in the nine independent studies reported across the eight publications included in the review. The studies employed established instruments with psychometric validation, including the Levenson Self-Report Psychopathy Scale (LSRP), the Psychopathic Personality Inventory–Short Form (PPI-SF), the Self-Report Psychopathy Scale III (SRP-III), and the Youth Psychopathic Traits Inventory (YPI). These measures enable the assessment of different components of psychopathy, including affective–interpersonal dimensions (Factor 1) and impulsive–behavioural dimensions (Factor 2), as well as characteristics such as emotional callousness, interpersonal manipulativeness, impulsivity, and antisocial tendencies. However, the instruments differ in their target populations, assessment purposes, and operationalisation of psychopathic traits. These differences should be considered when interpreting and comparing findings across studies.

In Brassard et al. [12], secondary psychopathy showed a positive association with the perpetration of mild psychological violence, whereas the corresponding association with primary psychopathy was weaker. In contrast, primary psychopathy showed a positive association with more frequent perpetration of severe psychological violence, whereas the corresponding association with secondary psychopathy was weaker. The reported associations differed according to the psychopathy dimension assessed and the severity of psychological violence. Similarly, Iyican and Babcock [10] found that Factor 1 (affective–interpersonal) of the PPI-SF was correlated with all forms of self-reported psychological violence among the men in the sample, whereas no corresponding associations were reported for Factor 2.

In Humeny et al. [18], psychopathic traits were assessed through participants’ reports of their abusive partners rather than through direct assessment of those individuals. Participants described their partners as displaying moderate levels of psychopathic traits, particularly interpersonal manipulativeness and an emotionally detached affective style associated with Factor 1. These traits were associated with a range of forms of violence within intimate partner relationships. Conversely, Plouffe et al. [11] found that Factor 2 was associated with greater severity in the perpetration of intimate partner violence in one of their studies. In their first study, both Factor 1 and Factor 2 were correlated with psychological and physical aggression, whereas bidirectional psychological aggression was stronger at lower levels of both psychopathy factors.

In Brazil and Forth [13], overall psychopathic traits assessed using the PCL:YV were associated with both emotional–verbal abuse and dominance–isolation. Similarly, Bui and Pasalich [17] found statistically significant indirect associations between insecure attachment and the perpetration of both face-to-face and cyber psychological violence through psychopathic traits.

Finally, Fontanesi et al. [14] assessed Dark Triad traits and reported a specific positive association between psychopathy and psychological abuse.

#### 3.6.2. Psychological Violence

Psychological violence was assessed in all nine independent studies included in the review (100%). To this end, widely validated instruments were employed, including the Conflict Tactics Scale–Revised (CTS-2), the Psychological Maltreatment of Women Inventory (PMWI), and the Multidimensional Measure of Emotional Abuse (MMEA). These measures enabled the examination of variables related to different manifestations of psychological violence, including verbal aggression, coercive control, humiliation, emotional manipulation, dominance–isolation, and intimidation.

In the study by Brassard et al. [12], psychological violence was classified into two distinct levels: mild and severe. Primary psychopathy showed a positive association with severe psychological violence. Brazil and Forth [13] likewise identified two principal dimensions of psychological violence: emotional–verbal abuse and dominance–isolation.

In the study by Bui and Pasalich [17], both face-to-face and cyber psychological violence showed similar associations with psychopathic traits and characteristics of borderline personality disorder. For their part, Fontanesi et al. [14] reported elevated levels of psychological violence, particularly in the form of restrictive intimidation and denigration. The study also revealed gender differences: women reported greater involvement in certain forms of abuse, whereas men obtained higher scores on the Dark Triad and on overall violence. Furthermore, dark personality traits, especially those related to psychopathy, were found to be associated with higher levels of psychological violence and greater control over one’s partner.

From the victims’ perspective, Humeny et al. [18] reported very high rates of emotional abuse and deception.

The study by Iyican and Babcock [10] also yielded a noteworthy pattern. Self-reported psychological violence among men showed stronger associations with Factor 1 psychopathy than women’s reports of male psychological violence.

Similarly, Thornton et al. [19] reported positive associations between psychopathic traits and psychological violence among both men and women in a university population.

Finally, Plouffe et al. [11] found that bidirectional psychological aggression was stronger at lower levels of Factor 1 and Factor 2 psychopathy.

#### 3.6.3. Relationship Between Subclinical Psychopathy and Psychological Violence

The relationship between subclinical psychopathy and psychological violence was examined in the nine independent studies reported across the eight publications included in the review. Associations between subclinical psychopathic traits and psychological violence were reported across the included studies. The strength and nature of these associations varied according to the type of sample, the instrument employed, and the form of violence assessed. Both affective–interpersonal dimensions (Factor 1) and impulsive–behavioural dimensions (Factor 2) were associated with psychological violence, with different patterns reported across studies.

Associations involving Factor 1 varied across studies. Brassard et al. [12] and Iyican and Babcock [10] reported associations between affective–interpersonal traits and forms of psychological violence involving control, manipulation, or intimidation. Humeny et al. [18] reported related patterns based on participants’ descriptions of their abusive partners’ psychopathic traits; these findings should therefore be interpreted in light of the informant-based assessment method. In some studies, primary psychopathy was associated with forms of psychological violence characterised by dominance, humiliation, or emotional intimidation.

By contrast, Factor 2 exhibits a more variable pattern. Brassard et al. [12] found a positive association between secondary psychopathy and mild psychological violence, whereas the corresponding association with severe psychological violence was weaker. Plouffe et al. [11] found that Factor 2 was associated with greater severity of intimate partner violence perpetration in one study. In their first study, Factor 2 was correlated with psychological and physical aggression, while bidirectional psychological aggression was stronger at lower levels of Factor 2 psychopathy.

Brazil and Forth [13] reported indirect associations between interparental violence and dominance–isolation and emotional–verbal abuse through psychopathic traits, after controlling for age and sample type. However, because the study was cross-sectional, these findings should not be interpreted as evidence of a temporal or causal mediation pathway. Similarly, Bui and Pasalich [17] reported indirect associations between insecure attachment and the perpetration of psychological violence through psychopathic traits. Given the cross-sectional design, these indirect associations should likewise not be interpreted as evidence of temporal or causal mediation.

Across samples, the reported patterns also differed according to participant characteristics and informant. In studies including exclusively male participants, associations between psychopathy and psychological violence included forms involving manipulation and control. Mixed-gender samples reported associations with different forms of psychological violence, including restrictive intimidation and denigration among women [14]. In studies focusing exclusively on women, participants reported associations between psychopathic traits attributed to their partners and different forms of psychological violence.

## 4. Discussion

This systematic review included nine independent studies reported across eight publications, all of which employed cross-sectional designs, to examine the association between subclinical psychopathy and psychological violence in intimate partner relationships. Overall, the findings suggested a positive association between psychopathic traits and multiple forms of psychological violence, although the strength and consistency of these associations varied across psychopathy dimensions, violence outcomes, samples, and analytical approaches. The evidence base was limited by the exclusive reliance on cross-sectional data, substantial heterogeneity in measurement instruments, and the predominant use of self-report measures. Therefore, while the findings provide preliminary support for an association between subclinical psychopathy and psychological violence, the overall level of confidence in causal and temporal interpretations remains limited.

Against this background, increasing attention has been directed towards understanding psychopathy beyond forensic populations. Scholarly interest has traditionally focused on prototypical psychopathy and violence; however, psychopathic traits are not confined to offender populations [27,28,29,30]. Accordingly, a progressive shift can be observed from categorical models focused exclusively on criminal psychopathy towards a dimensional conceptualisation, which facilitates an understanding of how psychopathic traits may manifest at subclinical levels within community and relational contexts.

In line with these findings, the studies included in the review consistently indicate that not all components of psychopathy contribute equally to the perpetration of psychological violence. In particular, traits associated with Factor 1, encompassing interpersonal and affective deficits such as emotional coldness, interpersonal manipulativeness, lack of empathy, and dominance, were generally associated with more severe and instrumental forms of psychological violence. Accordingly, behaviours such as coercive control, humiliation, emotional intimidation, and social isolation appear to be particularly closely linked to this psychopathic profile, as evidenced by the studies conducted by Iyican and Babcock [10] and Humeny et al. [18]. However, Brassard et al. [12] reported a more nuanced pattern, with primary psychopathy being associated with severe psychological violence and secondary psychopathy being associated with mild psychological violence, while the alternative psychopathy factor was not significantly associated with each respective outcome.

These findings are consistent with theoretical models that conceptualise psychopathy as a pattern characterised by the strategic use of interpersonal relationships for purposes of control and personal gain [30,31]. From this perspective, psychological violence may reflect forms of instrumental interpersonal control rather than solely reactive responses to conflict. However, this interpretation remains a theoretical explanation, as the studies reviewed did not directly assess the motivations or intentional processes underlying these behaviours.

By contrast, the role of Factor 2, which is more closely associated with an antisocial lifestyle and characterised by traits such as impulsivity and emotional dysregulation, exhibits a more heterogeneous pattern across the studies analysed. Thus, some studies have identified significant associations between this factor and forms of psychological violence such as hostile verbal expressions and emotional aggression [12,13]. However, the studies reviewed did not directly assess the motivational or regulatory processes underlying these behaviours, and therefore conclusions regarding their reactive nature cannot be drawn. However, these relationships appear to depend to a greater extent on the type of sample and the relational context. While Factor 2 assumes greater relevance in younger samples or populations experiencing higher levels of social vulnerability [11], its explanatory value appears to be more limited in community adult populations, where it has been more frequently associated with physical violence [18].

Another relevant finding concerns the indirect association involving psychopathic traits in the relationship between dysfunctional relational variables and psychological violence. In this regard, the study by Bui and Pasalich [17] reported a significant cross-sectional indirect association between insecure attachment and the perpetration of emotional abuse in both face-to-face and cyber contexts through psychopathic traits. This finding suggests that subclinical psychopathy may be associated with the link between pre-existing relational vulnerabilities and the perpetration of psychological violence; however, the cross-sectional nature of the data precludes conclusions regarding temporal or causal mechanisms.

Similarly, several of the studies reviewed highlight that psychological violence associated with subclinical psychopathy is not confined to clinical or forensic settings, but is also present in educational, community, and young dating populations. Findings obtained from both university samples [19] and adolescent and young adult populations [11,13] indicate that the association between psychopathic traits and psychological violence may also be observed in younger populations. These findings may inform future research on prevention in the context of young people’s romantic relationships.

On the other hand, although some studies report higher levels of psychopathic traits and violence among men [19], others indicate that certain specific forms of psychological violence, such as denigration and restrictive intimidation, were reported more frequently among women in particular samples [14]. However, such differences should be interpreted with caution, as they may reflect variations in measurement, reporting practices, sample characteristics, relationship context, or item interpretation rather than stable sex- or gender-related patterns. These findings suggest that the relationship between subclinical psychopathy and psychological violence cannot be interpreted in a unidimensional manner; rather, it is necessary to consider contextual, relational, and sex- and gender-related variables. It is noteworthy in this regard that the majority of research on psychopathy has focused on male populations, often overlooking the particular ways in which this construct may manifest as a function of gender [32,33]. Nevertheless, previous research has linked subclinical psychopathy to the perpetration of psychological violence within intimate partner relationships, with some studies reporting violence perpetration by both partners. However, the presence of bidirectional or reciprocal acts should not be interpreted as indicating equivalence in coercive control, severity, fear, harm, or power dynamics, dimensions that were not consistently assessed across the studies reviewed [28,34,35,36].

Taken together, the findings of the present systematic review suggest that psychopathic traits are associated with psychological violence in intimate partner relationships. These findings should be interpreted in light of the methodological limitations of the included studies.

First, the present systematic review was not prospectively registered in a protocol registration platform prior to the initiation of the review process. Consequently, no registration number or prospectively registered protocol date is available. Although the eligibility criteria, search strategy, study selection process, data extraction, and methodological assessment procedures are described in detail in the present manuscript, the absence of prospective registration limits the possibility of independently verifying whether these methodological and analytical decisions were established before the results of the included studies were known.

Second, all of the studies included adopted a cross-sectional design, which precludes the establishment of causal relationships and prevents the examination of temporal changes in psychopathic traits and psychological violence. As a result, it is not possible to determine whether psychopathic traits preceded psychological violence, whether relationship conflict and psychological aggression influenced the reporting of psychopathic traits, or whether reciprocal processes operated between these variables over time. Third, there is substantial heterogeneity in the assessment instruments employed for both psychopathy and psychological violence, which complicates the integration of findings across studies. Specifically, it is noteworthy that some studies utilised instruments designed to assess prototypical or criminal psychopathy in at-risk community samples with no criminal history and therefore outside forensic or correctional settings. Furthermore, the majority of studies relied on self-report measures, introducing potential biases associated with social desirability and differences in the perception of violence between perpetrators and victims. In Humeny et al. [18], psychopathic traits were assessed through participants’ reports about their abusive partners rather than through direct assessment of those individuals. This difference in the source of information should be considered when interpreting the findings, as informant-based assessments may introduce distinct sources of measurement bias.

In addition, the methodological quality of the included studies should be interpreted at the domain level rather than solely on the basis of the overall NOS-xs scores. Although all nine NOS-xs assessments obtained 7 out of 9 points, relevant methodological limitations were identified at the domain level, particularly regarding sample representativeness, measurement procedures, and the control of potential confounding factors. The diversity of recruitment settings and participant populations may also limit the comparability and generalisability of the findings across studies. These methodological characteristics should therefore be considered when interpreting the associations between psychopathic traits and psychological violence.

The restriction of the literature search to English-language publications represents an additional limitation. Although English is the predominant language of international scientific communication, this restriction may have introduced language bias and resulted in the omission of potentially relevant studies published in other languages. Future systematic reviews should consider multilingual searches to provide broader coverage of the international evidence.

The number and selection of bibliographic databases represent an additional limitation of the present review. Although the selected databases provided multidisciplinary coverage of the fields relevant to the review, the use of a broader range of bibliographic databases may have identified additional potentially relevant studies.

Finally, the absence of complementary search strategies, including reference-list screening, forward citation tracking, the grey literature, dissertations and theses, and direct contact with study authors, represents an additional limitation. Although the review was deliberately focused on published, peer-reviewed empirical research identified through predefined and reproducible searches of established bibliographic sources, these omissions may have resulted in the exclusion of potentially relevant published or unpublished evidence.

An additional limitation concerns the heterogeneity of participant populations and recruitment settings across the included studies. While most studies examined predominantly community-based samples, Brazil and Forth [13] included participants recruited from both secure custody facilities and community settings. Although this study met the predefined eligibility criteria, its mixed recruitment context may limit the comparability and generalisability of findings across studies.

Publication bias should also be considered when interpreting the findings of this review. It is therefore possible that studies reporting statistically significant associations between psychopathic traits and psychological violence were more likely to be published or emphasized than studies reporting null or non-significant findings. This possibility should be taken into account when interpreting the overall consistency and strength of the available evidence.

Despite the limitations identified, the present review synthesises the findings of the studies included in the search and contributes to the current understanding of the association between subclinical psychopathy and psychological violence within intimate partner relationships. The reviewed evidence suggests that psychological violence is an important interpersonal correlate of psychopathic traits across the populations represented in the included studies. From an applied perspective, these findings highlight the potential relevance of subclinical psychopathic traits to understanding psychological violence within intimate partner relationships. Although the available evidence does not establish the clinical utility of assessing these traits in prevention, screening, or intervention programmes, future research should examine whether their assessment may provide additional value for identifying risk and informing intervention approaches. This finding supports the existence of an association between subclinical psychopathic traits and several forms of psychological violence reported across the reviewed studies [28,31,37].

Therefore, identifying and understanding the psychological dynamics associated with subclinical psychopathy may contribute to advancing research on psychological violence within intimate partner relationships. However, elevated psychopathic-trait scores should not be interpreted as evidence that an individual has engaged in, or is likely to engage in, abusive behaviour. Prevention and intervention efforts should remain focused on observable behaviours and empirically supported risk indicators. Future longitudinal studies employing multimethod designs and diverse samples will be essential for clarifying the temporal relationships between psychopathic traits and psychological violence, while intervention research should examine whether incorporating knowledge of these traits can improve prevention or intervention outcomes.

## 5. Conclusions

The present systematic review examined studies published between January 2015 and March 2026 on the association between subclinical psychopathy and psychological violence within intimate partner relationships. Increasing attention to subclinical psychopathy, and its distinction from criminal psychopathy, has fostered a broader understanding of this construct, enabling researchers to examine how specific psychopathic traits may manifest within community populations and be associated with different forms of psychological violence. As demonstrated by the studies included in this review, psychopathic traits, including those linked to the affective–interpersonal dimension, were associated with behaviours involving coercive control, interpersonal manipulation, humiliation, and emotional intimidation, although the strength and consistency of these associations varied across outcomes and studies.

Despite the limitations identified, the findings contribute to a better understanding of the association between subclinical psychopathy and psychological violence within intimate partner relationships. Furthermore, these findings extend current knowledge of this phenomenon beyond clinical and forensic contexts by integrating the available evidence from community populations.

The findings highlight the potential relevance of subclinical psychopathic traits to understanding psychological violence within intimate partner relationships and provide a basis for further research into prevention and intervention. Future research should examine whether the assessment of subclinical psychopathic traits provides additional value for identifying risk and informing prevention and intervention strategies, and whether incorporating this information leads to improved outcomes. Such knowledge may contribute to the prevention of psychological violence and the promotion of more adaptive intimate partner relationships within the populations represented in the available evidence, although further research involving more diverse relationship contexts is needed.

## Figures and Tables

**Table 1:** Studies excluded after full-text assessment and specific reasons for exclusion.

Authors	Specific Reason for Exclusion
Blanchard & Fino [20]	Did not assess psychological violence or abusive behaviour in intimate relationships; focused on love styles and capacity to love.
2. Ferreiros & Clemente [21]	The study did not directly assess psychological violence or abusive behaviors in intimate relationships, focusing instead on dark personality traits, moral disengagement, and relationship behaviors.
3. Ireland et al. [22]	The study did not assess psychological violence or abusive behaviours in interpersonal relationships. It focused on developmental trauma and childhood abuse in relation to psychopathic traits.
4. Reidy et al. [23]	The study was a theoretical review and did not report original empirical data on the relationship between psychopathy and psychological violence.
5. Romero-Martínez et al. [24]	The study sample consisted exclusively of men convicted of intimate partner violence who were participating in a mandatory intervention programme.
6. Waller et al. [25]	The study did not assess psychological violence or interpersonal abusive behaviours.
7. Welner et al. [26]	The study was a theoretical and instrument-development article and did not include an empirical assessment or measure subclinical psychopathy or psychological violence using validated instruments.

**Table 4:** Risk of Bias Assessment of the Included Studies Using the Newcastle–Ottawa Scale Adapted for Analytical Cross-Sectional Studies (NOS-xs).

Study	Representativeness of the Study Sample	Sample Size	Assessment of the Exposure(s)	Assessment of the Outcome(s)	Adjustment for Confounder(s)	Assessment of Confounder(s)	Overall NOS-xs Score
Brassard et al. [12]	0/1	1/1	2/2	2/2	1/2	1/1	7/9
2. Brazil & Forth [13]	0/1	1/1	2/2	1/2	2/2	1/1	7/9
3. Bui & Pasalich [17]	1/1	1/1	1/2	1/2	2/2	1/1	7/9
4. Fontanesi et al. [14]	1/1	1/1	1/2	1/2	2/2	1/1	7/9
5. Humeny et al. [18]	0/1	1/1	1/2	2/2	2/2	1/1	7/9
6. Iyican & Babcock [10]	0/1	1/1	1/2	2/2	2/2	1/1	7/9
7. Plouffe et al. [11]—Study 1	0/1	1/1	1/2	2/2	2/2	1/1	7/9
8. Plouffe et al. [11]—Study 2	0/1	1/1	1/2	2/2	2/2	1/1	7/9
9. Thornton et al. [19]	1/1	1/1	1/2	1/2	2/2	1/1	7/9

Note: Values represent the number of stars awarded for each NOS-xs item. The total score ranges from 0 to 9 stars, with higher scores indicating better methodological quality according to the NOS-xs criteria.

## Data Availability

No new data were created or analysed in this study. Data sharing is not applicable to this article.

## Supplementary Materials

The following supporting information can be downloaded at https://www.mdpi.com/article/10.3390/healthcare14203499/s1, Table S1: PRISMA checklist; Table S2: Database-specific search strategies, search fields, filters, and search date.
