# Supplementary material for: Psychopathic Traits and Psychological Violence in Intimate Partner Relationships: A Systematic Review of Observational Studies
Source: Healthcare (Basel). 2026 Oct 18;14(20):3499. doi: 10.3390/healthcare14203499 (PMC13704379; doi:10.3390/healthcare14203499)
Supplement: Supplementary file 1 [file healthcare-14-03499-s001.zip › healthcare-4579417-Supplementary Table S2.pdf]

Supplementary Table S2. Database-specific search strategies, search fields, filters, and search date.

Database	Search strategy	Search field	Filters/limits	Search date
Web of Science	(“integrated psychopathy” OR “subclinical psychopathy” OR “psychopathic traits”) AND (“psychological violence” OR “psychological abuse” OR “psychological maltreatment”)	Topic (TS)	Publication year: January 2015–March 2026	12 April 2026
Scopus	(“integrated psychopathy” OR “subclinical psychopathy” OR “psychopathic traits”) AND (“psychological violence” OR “psychological abuse” OR “psychological maltreatment”)	Article title, Abstract, Keywords	Publication year: January 2015–March 2026	12 April 2026
PsycINFO (EBSCOhost)	(“integrated psychopathy” OR “subclinical psychopathy” OR “psychopathic traits”) AND (“psychological violence” OR “psychological abuse” OR “psychological maltreatment”)	All Fields	Publication year: January 2015–March 2026	12 April 2026
ScienceDirect	(“integrated psychopathy” OR “subclinical psychopathy” OR “psychopathic traits”) AND (“psychological violence” OR “psychological abuse” OR “psychological maltreatment”)	All fields / general search	Publication year: January 2015–March 2026	12 April 2026
